# Comparison of adsorption of selected antibiotics on the filters in continuous renal replacement therapy circuits: in vitro studies

**DOI:** 10.1007/s10047-019-01139-x

**Published:** 2019-10-20

**Authors:** Dariusz Onichimowski, Hubert Ziółkowski, Krzysztof Nosek, Jerzy Jaroszewski, Elżbieta Rypulak, Mirosław Czuczwar

**Affiliations:** 1 Department of Anaesthesiology and Intensive Therapy, Faculty of Medicine, University of Warmia and Mazury, Al. Warszawska 30, 11-082 Olsztyn, Poland; 2 Department of Pharmacology and Toxicology, Faculty of Veterinary Medicine, University of Warmia and Mazury, Oczapowskiego 13, 10-719 Olsztyn, Poland; 3 Department of Pharmacology and Toxicology, Faculty of Medicine, University of Warmia and Mazury, Al. Warszawska 30, 10-082 Olsztyn, Poland; 4 2nd Department of Anaesthesiology and Intensive Therapy, Medical Univeristy of Lublin, Staszica 16, 20-081 Lublin, Poland

**Keywords:** Vancomycin, Gentamycin, Ciprofloxacin, Tigecycline, Continuous renal replacement therapy

## Abstract

The aim of this study was to assess the adsorption of selected antibiotics: vancomycin, gentamicin, ciprofloxacine and tigecycline in an experimental continuous veno-venous hemofiltration circuit with the use of both polyethyleneimine-treated polyacrylonitrile (PAN) and the polysulfone (PS) filter membranes. The crystalloid fluid dosed with one of antibiotic was pumped from a reservoir through a hemofiltration circuit (with PAN or PS membrane) and back to reservoir. All ultrafiltrate was also returned to the reservoir. During the procedures samples were collected from the post-hemofilter port at 5, 15, 30, 45, 60, 90, and 120 min. To determine spontaneous degradation of the antimicrobials, an additional bag with each study drug was prepared, which was not attached to the hemofiltration circuit. The samples from these bags were used as controls. In the case of vancomycin, gentamycin and tigecycline there was a statistically significant decrease in the drug concentration in the hemofiltration circuit in comparison to the control for PAN membrane (*P* < 0.05, *P* < 0.001, *P* < 0.001, respectively). In the case of ciprofloxacine adsorption was reversible and the drug concentrations increase to achieve the initial level for both membranes. Our studies indicated that a large portion of the administered dose of antibiotics may be adsorbed on a PAN membrane. In the case of gentamicin and tigecycline this amount is sufficiently big (over 90% of the administered dose) to be of clinical importance. In turn, adsorption on PS membranes is clearly lower (up to 10%) and may be clinically unimportant.

## Introduction

Recently an increase in the incidence of acute renal failure in patients of intensive therapy units has been observed. This contributes to the growing use of continuous renal replacement therapy (CRRT) [1]. CRRT may significantly affect the clearance of antimicrobial drugs, in some situations considerably accelerating their elimination. An enhanced clearance of an antibiotic may lead to the decrease in the drug concentration in the blood to subtherapeutic levels, making it impossible to successfully treat infections or septic shock. The rate of antibiotic elimination during CRRT is influenced by the intensity of the procedure (dialysis dose) and by the surface of the filter used [1, 2]. This elimination occurs not only through convection and diffusion but also through adsorption on the filter membrane of the CRRT circuit [3]. The intensity of the adsorption depends on the type of material from which the filter membrane is made [4]. For some antibiotics the filter elimination may be the main mechanism of its elimination. This mechanism is of greatest significance in the case of aminoglycosides, glycopeptides, fluoroquinolones, and polymyxins [5], although such data for glycylcyclines are lacking. The literature on the adsorption of antibiotics on the CRRT system filters is scarce. In the available studies for polyacrylonitrile (PAN) filters, the percentage of the loading dose bound in the filter may reach from 30% (vancomycin, levofloxacin) to as much as 90% (colistin) [5]. The number of reports on the adsorption on polysulfone (PS) membranes is smaller than that on PAN, but these membranes also can adsorb even 50% of the administered drug dose, as has been proven for tedizolid [6]. In recent years the manufacturer of AN69HF PAN membranes covered the membranes with polyethyleneimine (PEI) to increase their biocompatibility [7, 8]. The abovementioned intervention results in a decreased negative charge of the membrane, from − 70 mV zeta potential to − 15 mV, which may influence the adsorption of positively charged antibiotic molecules such as aminoglycosides [7, 9]. The majority of studies on the adsorption of gentamicin and vancomycin on CRRT filter membranes were conducted with the use of non-PEI-treated PAN membranes, therefore, there is an urgent need to assess the impact of the modification of the filter membrane on the antimicrobial binding. Moreover, over the past few years the surface area of the CRRT filters significantly increased from approximately 0.6–0.9 to 1.2–1.5 m^2^, which might influence the magnitude of drug adsorption. Currently there are no studies on the adsorption of ciprofloxacin and tigecycline. The reasons to believe that ciprofloxacin can be adsorbed on filter membranes is because it represents the same class as levofloxacin, which is known to undergo adsorption; and it is believed that tigecycline can be adsorbed on filter membranes because of its ability to adsorb to organic compounds not only through the mechanism of electrostatic attraction but also through metal bridging by forming ternary complexes [10, 11].

The aim of this study was to assess the in vitro adsorption of selected antibiotics representing various therapeutic groups (vancomycin, gentamicin, ciprofloxacin, and tigecycline) in an experimental CRRT circuit with the use of both PEI-treated PAN (AN69ST) and the PS filter membranes.

## Materials and methods

### In vitro study system

The study assessed adsorption on two types of membranes: AN69ST membrane filter (Gambro, France) with the surface of 1.5 m^2^ and PS membrane—AV 1000 filter (Fresenius Medical Care, Germany) with the surface of 1.8 m^2^ in a one-compartment model using the circuit for continuous veno-venous hemofiltration (CVVH). The adsorption was examined by means of a device used in clinical conditions (Multifiltrate, Fresenius Medical Care, Germany) with a set of drains by the same provider. The type of filter used in the drain set was either AN69ST or AV 1000. The approximate total circuit volume (reservoir and set of drains together) was 5500 mL. Three study cycles were performed for every type of filter and antibiotic. Adsorption was examined in CVVH circuit, with the fluid flow rate of 100 mL/min and ultrafiltration rate of 500 mL/h.

**Fig. 1:**
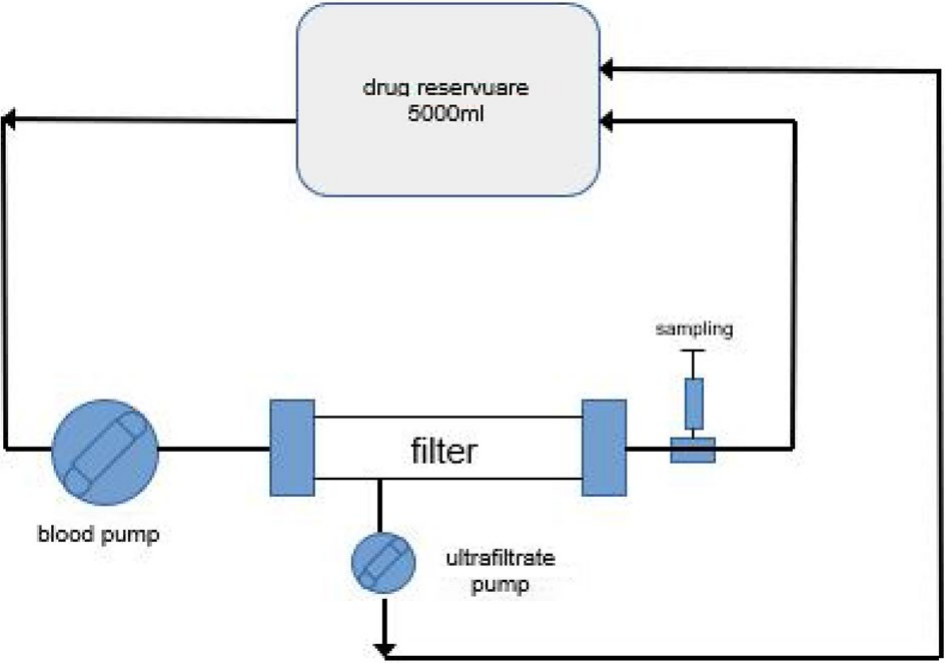
In vitro study system, representing the continuous veno-venous hemofiltration

Before the commencement of the study the circuit was filled with 0.9% saline solution (at the room temperature) without antibiotic. During the test the ultrafiltrate was continuously returned to the initial antibiotic solution (Fig. 1).

To assess adsorption, vancomycin (Edicin, Sandoz GmbH, Austria) was used at the dose of 1000 mg, gentamicin (Gentamycin; Krka, Slovenia) at the dose of 400 mg, ciprofloxacin (Ciprofloxacin Kabi; Fresenius Kabi, Germany) at the dose of 400 mg, and tigecycline (Tygacil; Pfizer Limited, Great Britain) at the dose of 100 mg. The drugs were dissolved in 5000 mL of crystalloid solution buffered to pH 7.4 by means of bicarbonates (Multibic K2; Fresenius Medical Care, Germany). The samples for the assessment of initial antibiotic concentration were obtained at baseline (0 min) from the drug-containing replacement solutions. During the experimental procedures, samples were collected from the post-hemofilter port at 5, 15, 30, 45, 60, 90, and 120 min. To determine spontaneous degradation of the studied antimicrobials, an additional bag with each study drug was prepared, which was not attached to the CVVH circuit. The samples from these bags were obtained at the same time as during the experimental procedure and were used as controls, following the mathematical correction of the dilution with CVVH circuit volume. Every study cycles were performed at the room temperature.

The samples were frozen immediately after the collection at − 80 °C and were sent to the laboratory for the determination of the drug concentrations.

### Drug analysis

To determine vancomycin and gentamicin concentrations in the collected samples, the ARCHITECT iVancomycin and ARCHITECT iGentamicin in vitro chemiluminescent microparticle immunoassay for the quantitative measurement of vancomycin and gentamicin, respectively on the ARCHITECT iSystem with STAT protocol capability (ABBOT, Germany) were used. The procedure was performed according to the manufacturer’s recommendations. The calibration ranges for vancomycin and gentamicin were 0.0–100.0 μg/mL and 0.0–10.0 μg/mL, respectively. The limit of detection for vancomycin was ≤ 3.0 μg/mL and gentamicin was ≤ 0.3 μg/mL.

The ciprofloxacin concentrations in collected samples were determined using fully validated liquid chromatography (LC) with fluorescent detection analytical method for enrofloxacin described previously by Ziółkowski et al. with minor modifications [12]. The modifications consisted in changing the excitation wavelength of light and emission.

Wavelength, which amounted to 280 nm and 453 nm, respectively (in place of 300 nm and 448 nm, respectively, used in enrofloxacin determination). Also the volume of added standards was changed from 25 to 10 µL. Because the ultrafiltrate was the matrix, no extraction procedure was carried out. The calibration curve ranged from 5 to 90 μg/mL and the limit of detection was 0.002 μg/mL.

The tigecycline concentrations in collected samples were determined using fully validated LC with tandem mass spectrometry detection analytical method described previously by Jasiecka-Mikołajczyk and Jaroszewski with minor modifications, which were shorter column (50 mm instead of 150 mm) and tigecycline-d9 as the internal standard (instead of minocycline) [13]. The calibration curve ranged from 0.1 to 100 μg/mL and the limit of detection was 0.01 μg/mL.

The total adsorption of the drug was calculated from the decrease of its concentration in the solution. The initial volume of the bag with solution was 5000 mL. After it was attached to the CVVH circuit, the total volume of the solution was 5500 mL (the volume of the bag with the drug + the volume of fluid filling the drain system and the filter). Therefore, the total adsorption of the antibiotic on the filter membrane was calculated from the formula:

$${\text{Drug}}\,{\text{adsorption}} = {C_0}\,(\upmu {\text{g/mL}}) \times 5000\,{\text{mL}} - {C_{120}} \times 5500\,{\text{mL}},$$

where *C* stands for concentration.

The total adsorption of the drug was calculated only for the results that were statistically significant.

The total clearance of the drugs were calculated from the ratio of area under time concentration curve and total dose.

### Statistical analysis

In statistical analysis, the mean standard deviation (± SD) concentration values between the control and different filters were compared. Additionally the differences between particular time points in the same filter and between different filters were analyzed. Statistical analysis was performed using one-way analysis of variance with Newman–Keuls multiple comparison test and two-tailed, unpaired Student’s *t* test (Graph Pad Prism 3.1; Graphpad Software, San Diego, CA, USA). *P* < 0.05 was considered statistically significant.

## Results

The analysis of the studied drugs’ concentrations in the control solutions revealed no spontaneous degradation in any case. The concentrations after 120 min did not differ statistically from the initial concentrations for all studied antibiotics. These results confirmed that none of the studied antimicrobials underwent a spontaneous degradation in the replacement solution used in the experimental CRRT circuit in this study.

**Fig. 2:**
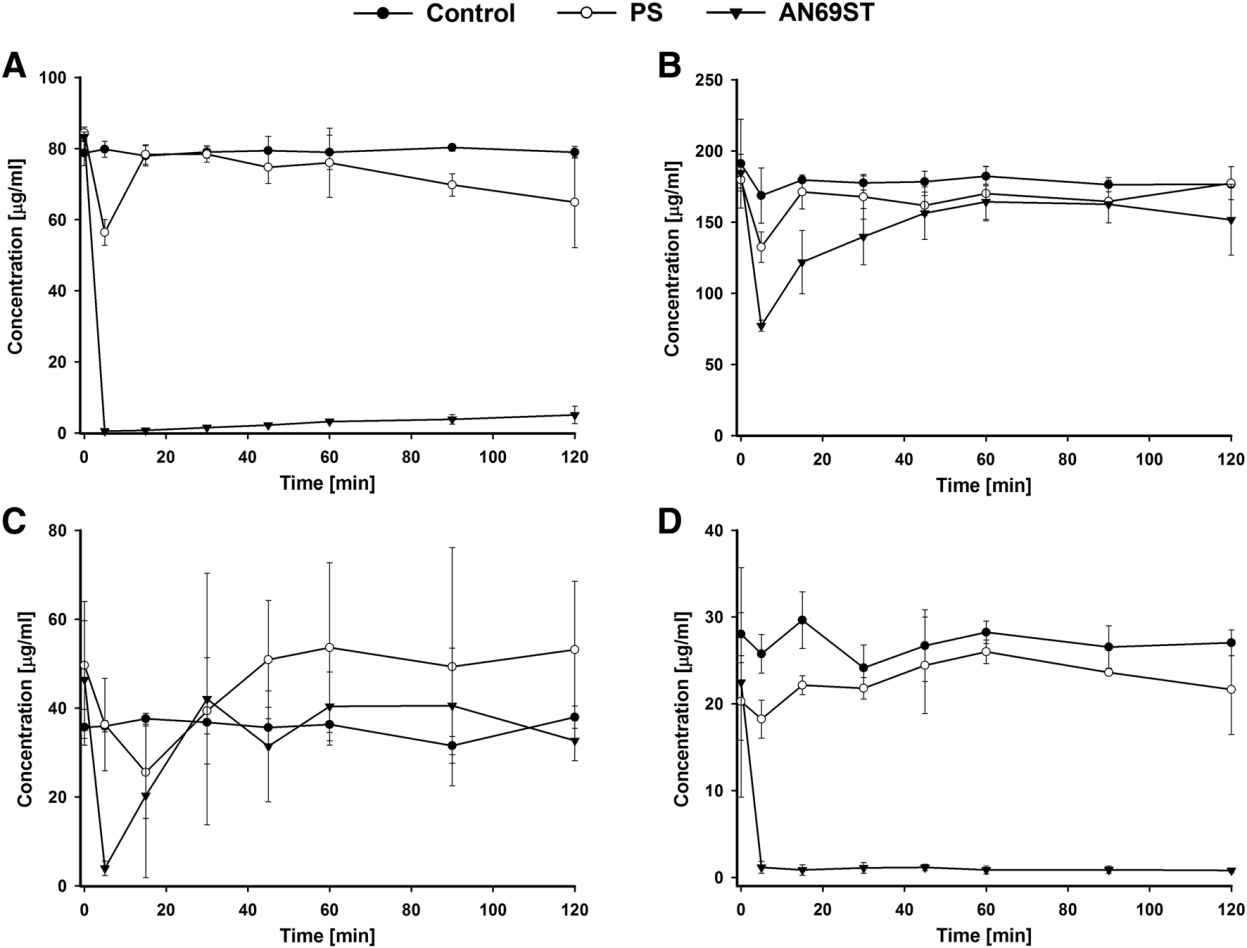
Concentrations (± SD, *n* = 3) of gentamycin (**a**), vancomycin (**b**), ciprofloxacin (**c**) and tigecycline (**d**) at different sampling times during the adsorption study. PS—concentrations in continuous veno-venous hemofiltration (CVVH) circuit with polisulfone membrane; AN69ST—concentrations in CVVH circuit with polyacrylonitrile treated polyethyleneimine membrane; control—concentrations in reservoir without CVVH after mathematical correction of the dilution with CVVH circuit volume

For gentamicin, a greater statistically significant adsorption was observed for AN69ST membrane in compared to PS membrane (*P* < 0.001). When compared with the control group, a statistically significant decrease in concentrations occurred only for AN69ST (*P* < 0.001) group. The lowest concentration of the drug for the PS membrane was observed in the fifth minute of the experiment, and later it remained on a relatively steady, high level until 120 min into the experiment (Fig. 2a). In the case of the AN69ST membrane, the drug concentration decreased dramatically as early as 5 min into the experiment and remained on a relatively steady, low level until the end of the experiment. The adsorption of antibiotic on the AN69ST membrane was 430.27 mg.

In the case of vancomycin there was a statistically significant decrease in the drug concentration in the CVVH circuit in compared to the control only for AN69ST membrane (*P* < 0.05). Substantial adsorption was noted on membranes of both types during the first 15 min of the experiment, with the highest adsorption observed in the fifth minute (*P* < 0.05) following the initiation of the filter perfusion (Fig. 2b). The statistically significant differences for both membranes. Subsequently, in 120 min into the study, the drug concentrations increased to 98.66% and 82.08% of the initial values for PS and AN69ST membranes, respectively. The adsorption of antibiotic on AN69ST membrane was 181.88 mg.

A significant decrease (*P* < 0.05) in ciprofloxacin concentration was observed in minute 5 (on an AN69ST membrane) and minute 15 (on a PS membrane) (Fig. 2c) compared to the initial values. The adsorption proved reversible, and the drug concentrations for both membranes increase to achieve the initial level for PS membranes (70.47% for AN69ST membrane in minute 120 of the experiment). Hence, no statistical difference was found, when considered in hole profile, for both membranes in comparison to control.

In the case of tigecycline, a significant decrease (*P* < 0.001) in the concentrations was observed only when AN69ST membrane was used (*P* < 0.001) (Fig. 2d). For the PS membrane the concentration after 120 min did not differ from the initial concentration. In the circuit with AN69ST filter, the concentration decreased in minute 5 to 5.2% of the initial concentration and remained at the similar level until the end of the experiment. The total calculated adsorption of the antibiotic on the AN69ST filter membrane was 119.17 mg.

**Fig. 3:**
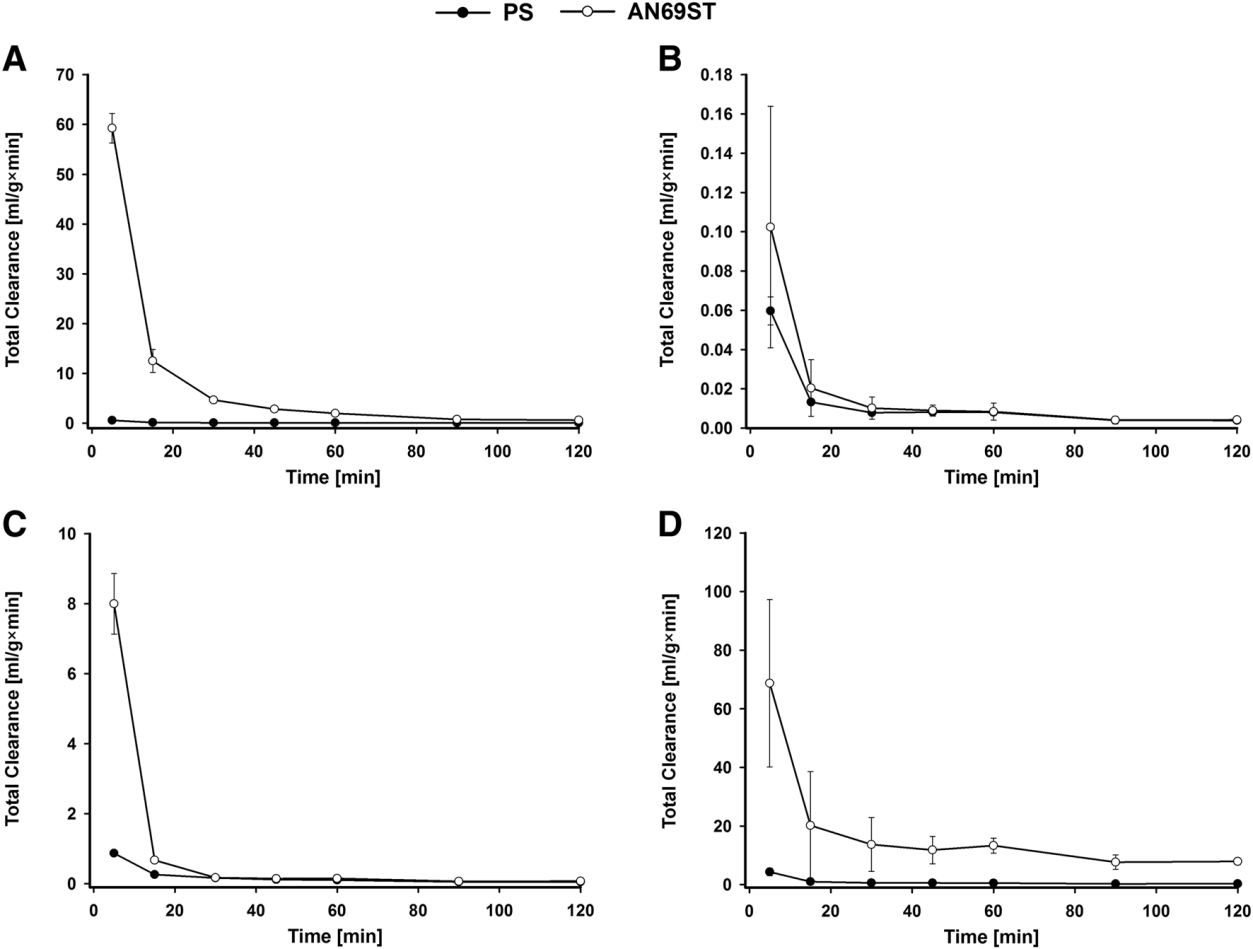
Total clearance (± SD, *n* = 3) of gentamycin (**a**), vancomycin (**b**), ciprofloxacin (**c**) and tigecycline (**d**) during the adsorption study. PS—concentrations in continuous veno-venous hemofiltration (CVVH) circuit with polisulfone membrane; AN69ST—concentrations in CVVH circuit with polyacrylonitrile treated polyethyleneimine membrane

The clearances of studied antimicrobials are presented in Fig. 3. Except vancomycin, the clearance was significantly higher for studied drugs when the AN69ST filter was used. For gentamycin the value of this parameter was higher at each time point and for ciprofloxacin in the first 15 and after 60 min. In turn, for tigecycline the clearance was significantly higher after 5, 60, 90 and 120 min.

## Discussion

Optimum antibiotic dosage is the primary criterion maximizing the survival chances of critically ill septic patients [14]. A clear relationship has been demonstrated between pharmacokinetic variables and favourable outcome. In septic patients treated with CRRT, these variables are likely to be affected by extracorporeal clearance. While the blood flows through the filter, adhesion of the antibiotic to the filter occurs, which results in the decrease of its concentration in the blood. In our study, the drug concentration of vancomycin in the blood decreased significantly only for the AN69ST membrane. The total adsorption of the drug was 181.88 mg. Similarly, in a study conducted by Tian et al., who compared the adsorptions on poliamide, PS, and AN69 (PAN) membranes, the adsorption on the latter was the greatest, being 10.08 mg, which constituted 28% of the administered dose [4]. When compared with Tian’s et al. study, the total amount of vancomycin adsorbed on the PAN membrane in our study was considerably greater. This could be because of the fact that the initial drug concentration in our study was nearly fourfold higher. In our study we assessed the AN69ST membrane, it means PEI-treated PAN. PEI surface changes the adsorption properties of the membrane, increasing the adhesion of some complement components and decreasing the negative charge of the membrane. Both of these phenomena could have affected the adsorption in our study. Also, the surface of the filter was greater (0.6 m^2^ in Tian’s group study vs. 1.5 m^2^ in our study). A smaller absolute amount of the drug absorbed in Tian’s group study could also be because of the fact that blood-crystalloid mixture was used in that study and the portion of the drug bound to the proteins did not undergo adsorption [3, 15]. Quale’s group study (1992) [16] assessed in vivo vancomycin adsorption during hemodialysis lasting 3.5 h, using a PAN filter of surface size similar to that used in our study (1.7 m^2^) and in vitro, using normal saline and fresh frozen plasma. In the in vivo study, a decrease by 32% in the drug concentration in plasma was observed. In the in vitro study, it was demonstrated that the drug adsorption on the membrane occurs both with and without proteins being present and with the protein presence increasing drug adsorption. This fact can be of clinical significance because it indicates that, together with the increasing time of filter use, additional adsorption may occur in the filter not directly on the membrane but on the proteins that deposited on it. For PS the adsorption of vancomycin in our study was not statistically significant, unlike in Tian’s group study [4]. In Tian’s group study, blood-crystalloid mixture was used, whereas in our study crystalloid solution was used [4]. This fact indicates a significant impact of proteins on the increased adsorption of antibiotics on the filters.

In our study, we found significant adsorption of gentamicin on the AN69ST membrane. The adsorption was fast and irreversible and constituted more than 90% of the dose used. The result we obtained for the AN69ST membrane correlated with the study conducted by Lam et al. [17] for the PAN membrane, in which gentamicin adsorption reached over 90%, was fast (up to 20 min), and was irreversible. Similar results were obtained by Tian’s group [9, 15] for amikacin. Gentamicin, like all aminoglycosides, has a molecule of a positive charge and, because of that, it is characterized by high adhesion to negatively charged membranes. PAN, AN69, and Multiflow 60 membranes, as used in Tian’s group study [9, 15] and Lam’s group study [17], respectively, are charged negatively at − 70 mV. In our study, we used PEI-treated PAN membranes, which resulted in decreasing their negative potential to − 15 mV [7]. Despite that fact, gentamicin adsorption was still very high and almost all the drug underwent adsorption. This phenomenon could be explained by the fact that, during the convection through the membrane to the filtrate compartment, the drug comes in contact with all the membrane thickness and not only with its surface. The charge of the PS membrane is − 51 mV, so it is comparable to those of PAN membranes; nevertheless, in our study, on membrane of the same type, only one-fourth of the dose administered underwent adsorption, not reaching the level of statistical significance [7]. This suggests that in gentamicin adsorption not only an electrostatic mechanism is involved, but also the membrane structure (solid or porous) may play a role. Gentamicin belongs to the group of concentration-dependent antibiotics. In the case of both membranes adsorption can significantly contribute to the lowering of peak concentration and thus preclude reaching therapeutic levels. Because of the fact that aminoglycosides should be administered in a single dose once a day, it seems reasonable to consider a break in CRRT, especially in the case of PAN membranes for the time of antibiotic administration so that it could reach the right concentration in the blood and a therapeutic effect. It is also suggested by other authors that aminoglycoside adsorption on the filter membrane during CRRT should be considered in their administration regime in critically ill patients [3, 5, 15, 18].

Data concerning the adsorption of quinolones are scarce and focus mainly on levofloxacin [19, 20]. In our study, we found no adsorption that would be statistically important in any filters. We observed, however, the trend toward a higher absorption on the AN69ST membrane. In both cases in the early phase of the experiment, significant adsorption was seen, but it proved reversible. In Tian’s group study adsorption of levofloxacin on the PAN membrane was also reversible and reached a low value (1.2% of the dose used) [20]. Pharmacokinetic studies of quinolones during CRRT conducted by other researchers show significant extrarenal elimination of ciprofloxacin. It is possible that, in some part, this elimination could be caused by adsorption on the filter membranes, particularly in the case of PAN membranes [5, 21, 22].

Tigecycline is a lipophilic antibiotic with a small molecule and a large volume of distribution, particularly in critically ill patients. Recently published papers suggest that the doses should be increased to improve treatment efficacy in comparison to the currently recommended dose [23–25]. A relatively high degree of protein binding (71–78%) and a large volume of distribution (*V*_ss_ > 900 L) theoretically decreases the elimination of this antibiotic through CRRT [26]. To date there have been no studies on the adsorption of tigecycline on the filter membranes used for CRRT. Broeker et al. [27] report that during the pharmacokinetic assessment of tigecycline during CRRT using PS filters in a group of 11 patients, one of the patients experienced a time delay in the effluent concentrations of tigecycline, which may have been caused by adsorption losses. In our study, a statistically significant, substantial adsorption was observed (more than 95%) for the AN69ST membrane. Tigecycline is a concentration-dependent antibiotic with a time component (AUC24/MIC), so the adsorption of this drug on filter membrane may effectively preclude reaching therapeutic concentrations with the currently recommended dosage. It seems necessary to conduct further pharmacokinetic tests in critically ill patients treated with CRRT with the consideration given to particular membrane types. Because of the fact that 59% of tigecycline is excreted with bile and faeces, adding an additional route of elimination (i.e. CRRT) may cause a decrease in drug concentration in blood, even below the values obtained in patients without renal failure [25].

A limitation of our study was the fact that the study was conducted in the protein-free crystalloid solution. Many authors use mixtures of crystalloids with blood or plasma [4, 16, 17] or full bovine blood [6] in their studies. The influence of proteins on antibiotic adsorption on membranes may be widely varied [3, 7, 16]. On the one hand, for drugs, which strongly bind to albumin, the amount of the drug absorbed may decrease. On the other hand, though, together with proteins depositing on the filter membrane, this amount may significantly increase. Hence, it seems necessary to conduct further tests in solutions containing proteins with a longer experiment time so as to reach clinical times allowed for filter use, that is 24 h. Another limitation is the fact that the study was conducted using liquids at room temperature which could affect the ability of adsorption. This fact was caused by the lack of a heating system guaranteeing maintaining a constant temperature in device used. However, it should be noted that in experiments carried out by other authors [16, 19, 28] saline at room temperature was also used.

## Conclusion

Reaching the therapeutic concentrations for antibiotics in the blood of patients treated for sepsis or septic shock is of key importance for their survival. Implementation of CRRT into their treatment significantly affects the elimination of antibiotics from their blood. Apart from convection and diffusion, an important mechanism of antibiotic elimination seems to be adsorption on filters, particularly when PAN membranes are used. Together with the growing surfaces of the filters used, the phenomenon seems to become increasingly significant. Our in vitro studies indicated that a large portion of the administered dose—not only of hydrophilic antibiotics (vancomycin, gentamicin) but also of lipophilic ones (tigecycline) may be adsorbed on a PAN membrane. In the case of gentamicin and tigecycline this amount is significant enough (more than 90% of the administered dose) to be of clinical importance. In turn, adsorption on PS membranes is clearly lower (up to 10%) and may not be clinically important. A significant decrease in gentamicin and tigecycline concentrations for AN69ST filters found in our study dictates the need to take into consideration the type of membrane used during CRRT. This may require corrections in the recommendations for the dosage of antibiotics, changes in administration routes (depending on the type of the used membranes), and taking into account the moment of a new filter insertion. Obviously, developing new recommendations requires further research in clinical conditions. We should possibly expect such information from CRRT technology suppliers. For tigecycline, the introduction of therapeutic drug monitoring might prove as beneficial as it is for gentamicin and vancomycin.
